# Relevance of sonography for botulinum toxin treatment of cervical dystonia: an expert statement

**DOI:** 10.1007/s00702-014-1356-2

**Published:** 2014-12-30

**Authors:** Axel Schramm, Tobias Bäumer, Urban Fietzek, Susanne Heitmann, Uwe Walter, Wolfgang H. Jost

**Affiliations:** 1 Department of Neurology, University of Erlangen, Erlangen, Germany; 2 Department of Movement Disorders and Neuropsychiatry, University of Lübeck, Lübeck, Germany; 3 Department of Neurology and Clinical Neurophysiology, Schön Klinik München Schwabing, Munich, Germany; 4 Department of Neurology, Deutsche Klinik für Diagnostik, Wiesbaden, Germany; 5 Department of Neurology, University of Rostock, Rostock, Germany; 6 Department of Neurology, University of Freiburg, Breisacher Str. 64, 79106 Freiburg, Germany

**Keywords:** Cervical dystonia, Botulinum toxin, Sonography, Ultrasound

## Abstract

Botulinum neurotoxin A (BoNT A) is the first-line treatment for cervical dystonia. However, although BoNT A has a favorable safety profile and is effective in the majority of patients, in some cases the treatment outcome is disappointing or side effects occur when higher doses are used. It is likely that in such cases either the target muscles were not injected accurately or unintended weakness of non-target muscles occurred. It has been demonstrated in clinical trials for spastic movement disorders that sonography-guided BoNT A injections could improve treatment outcome. As the published evidence for a benefit of sonography-guided BoNT injection in patients with cervical dystonia is scarce, it is the aim of this review to discuss the relevance of sonography in this indication and provide a statement from clinical experts for its use. The clear advantage of sonography-guided injections is non-invasive, real-time visualization of the targeted muscle, thus improving the precision of injections and potentially the treatment outcomes as well as avoiding adverse effects. Other imaging techniques are of limited value due to high costs, radiation exposure or non-availability in clinical routine. In the hands of a trained injector, sonography is a quick and non-invasive imaging technique. Novel treatment concepts of cervical dystonia considering the differential contributions of distinct cranial and cervical muscles can reliably be implemented only by use of imaging-guided injection protocols.

## Introduction

Botulinum neurotoxin A (BoNT A) is the first-line treatment for cervical dystonia (CD) and focal spasticity (Simpson et al. 2008, Truong and Jost 2006). The efficacy and safety of BoNT A has been demonstrated in numerous clinical studies leading to licensed use in many countries worldwide (Costa et al. 2005; Kamm and Benecke 2011; Simpson et al. 2008; Ade-Hall and Moore 2000; Truong and Jost 2006; Hallett et al. 2013; Esquenazi et al. 2013).

Several studies (at least for upper and lower limbs) showed a low accuracy of muscle localization when using anatomical landmarks only (Chin et al. 2005; Molloy et al. 2002; Schnitzler et al. 2012; Yang et al. 2009; Henzel et al. 2010). The accuracy of manual needle placement was not acceptable for the majority of target muscles. In consequence, it was concluded from these studies that ultrasound (US) could be considered as a valuable adjunct for muscle localization in patients with spasticity and that US can improve accuracy of BoNT A placement. However, to our knowledge, no studies have addressed the accuracy of needle placement in cervical muscles up to now.

With US guidance muscles can be targeted quickly and reliably by a non-invasive technique. US is of special value as the injected muscles differ in size, and deep-seated muscles require a different approach compared to superficial muscles (Fietzek et al. 2010; Gervasio et al. 2011). The US technique is already widely applied in neuropediatric care. For the treatment of spasticity injection control by US guidance has been recommended in several consensus statements (e.g., Wissel et al. 2011; Heinen et al. 2010). An improved clinical outcome could be demonstrated for US-guided BoNT A injections in children with cerebral palsy (Kwon et al. 2010; Py et al. 2009) and adults with spasticity after stroke compared to manual needle placement in two randomized controlled trials (Picelli et al. 2012a, 2013). In the lower limb, one of these studies (Picelli et al. 2012a) showed a trend for superiority of US guidance compared to electrical stimulation. In another study, neither manual needle placement nor electrical stimulation showed complete accuracy when compared to US (Picelli et al. 2012b).

In cervical dystonia, most clinical studies investigated the application of electromyography (EMG). In these studies, EMG was used not only for injection guidance but as well for muscle selection. Nevertheless, a recent review did not show an overall better outcome compared to clinical muscle selection and anatomical needle placement (Nijmeijer et al. 2012).

For the application of US guidance in cervical dystonia, only very few publications of non-controlled studies or case reports in a low number of patients are available which refer mainly to deep, difficult accessible muscles, e.g., longus colli or obliquus capitis inferior (OCI) (Bhidayasiri 2011; Fujimoto et al. 2012; Sung et al. 2007; Lee et al. 2009). Currently, there are no controlled comparative clinical studies of BoNT A injection according to anatomic landmarks versus sonography-guided injection in this indication. However, a recently published study although in a low number of patients with cervical dystonia provides evidence that US guidance of BoNT A injection in the sternocleidomastoid (SCM) muscle could reduce the rate of dysphagia (Hong et al. 2012). The authors concluded from their observations that recurrent dysphagia could reliably be eliminated by keeping the BoNT A solution within the SCM.

It is the aim of this review to discuss the application of US for injection control as well as the advantages and future perspectives of sonography-guided BoNT A injections in patients with cervical dystonia based on the available literature and our own experience.

### Differentiation from other techniques for injection control

Other techniques for injection control have been investigated in cervical dystonia, however several limitations have to be taken into account.

Although the use of computed tomography (CT) has been published in a small number of patients (Bhidayasiri 2011; Sung et al. 2007; Lee et al. 2009; Herting et al. 2004), its use is limited to a clinical setting where CT is accessible. Further on, CT is too expensive for frequent use in daily practice, it is not dynamic, and patients are exposed to radiation. Despite comparable anatomic precision of CT and US, the latter displays the major drawback for its use in long-term treatment of patients with cervical dystonia. The disadvantage of radiation exposure also applies to fluoroscopy (Glass et al. 2009) which requires multiple intramuscular injections of iodine-containing contrast medium.

Positron emission tomography (PET) has been used in two studies to identify hypermetabolic and presumably dystonic muscles, whereas injection was performed under CT or ultrasound guidance (Sung et al. 2007; Lee et al. 2009). PET therefore represents a rather diagnostic method, than a method for injection control.

Several studies have dealt with the use of EMG for BoNT A injection in cervical dystonia. All these studies should be interpreted with caution as EMG was simultaneously used for muscle selection. Nevertheless, only one randomized controlled trial showed better results for combined EMG selection and EMG guidance compared to clinical muscle selection with anatomical needle placement (Comella et al. 1992). A pooled analysis of 28 studies of which 17 used clinical evaluation to identify dystonic muscles and 11 used EMG for selection, and guidance showed better results for the EMG approach regarding pain reduction (−40.3 vs.−32.5 %). However, improvement was lower for EMG compared to clinical evaluation for clinical rating scales like the TSUI score (−31.9 vs.−43.7 %) (Nijmeijer et al. 2012). It has to be taken into account that positioning of the EMG needle in these approaches was performed according to anatomic landmarks. The placement of the EMG needle tip into a specific muscle is thereby almost impossible to verify, as selective voluntary activation of neck muscles is not possible and might be additionally superimposed by dystonic activity from adjacent muscles. EMG therefore serves more as a “functional” but not anatomical guidance as the assignment of EMG activity to specific muscles is flawed by the same anatomic inaccuracy as needle placement according to anatomical landmarks. Nevertheless, these shortcomings might be circumvented by combining EMG with ultrasound guidance (see also below). Finally “searching” for dystonic EMG activity is associated with additional discomfort and pain for the patient and extra costs for the EMG needle have to be taken into account.

In cervical dystonia, electrical stimulation is not feasible because for neck muscles the response to the stimulus is not specific enough.

Although CT (but not EMG) shows comparable anatomic precision as US, the latter is a non-invasive technique causing no additional discomfort for the patient nor exposing the patient to radiation or any further risks. US allows real-time visualization of muscles and the adjacent anatomical structures so that the entire injection procedure of BoNT A can be followed.

### Utility and advantages of US-guided injections

Availability of US in central Europe neurological clinics is high as neurovascular ultrasound is part of clinical routine diagnostics and almost all clinicians are familiar with the US machine on-site. Detailed recommendations on the technical requirements, procedure, and documentation of US-guided BoNT injection have been recently published (Walter and Dressler 2014).

**Fig. 1:**
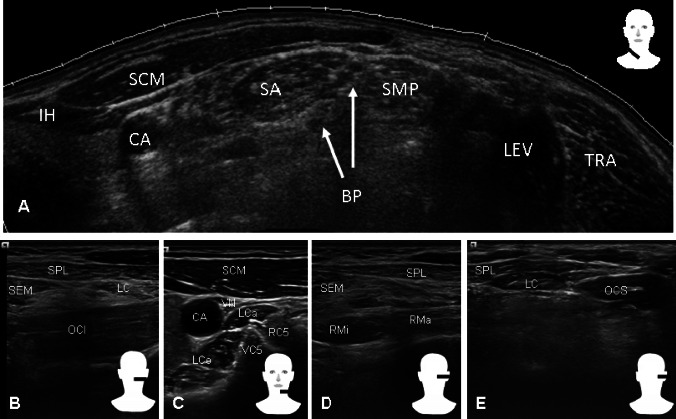
Sonographic appearance of frequently injected muscles (mainly **a**, **b**) and more difficult accessible, small, or deeper located muscles (mainly **c**, **d**, **e**). Muscles: *IH* infrahyoid, *SCM* sternocleidomastoideus, *SA* scalenus anterior, *SMP* scalenus medius posterior, *LEV* levator scapulae, *TRA* trapezius, *SEM* semispinalis capitis, *SPL* splenius capitis, *OCI* obliquus capitis inferior, *LC* longissimus capitis, *LCo* longus colli, *LCa* longus capitis, *RMi* rectus capitis posterior minor, *RMa* rectus capitis posterior major, and *OCS* obliquus capitis superior. Others are *CA* carotid artery, *BP* brachial plexus, *VC5* vertebra C5, *RC5* root C5, and *VN* vagus nerve

The obvious advantage of US is anatomic precision with accurate, safe, and optimal targeting of BoNT A injections in the affected muscles. With US, almost all neck muscles can be precisely visualized in the majority of patients. Figure 1 shows commonly injected muscles as the splenius capitis, sternocleidomastoideus, semispinalis capitis, and levator scapulae muscles (Fig. 1a, b), as well as more difficult accessible, small, or deeper located muscles like the longus colli, longus capitis, scalene, and obliquus capitis inferior and superior muscles (Fig. 1c, d, e). These neck muscles lie in close proximity to each other rendering false injections in adjacent muscles likely. Table 1 displays typical injection errors and summarizes the clinical experiences from the authors.

**Table 1:** Relevance of ultrasonography (US) for injection of muscles in cervical dystonia

Muscle	Relevance of US for muscle location	Relevance of US to avoid adverse reactions or injuries	Typical injection errors	Specific adjacent anatomical structures
Infra-/suprahyoid muscles	+++	+++		Several
Sternocleidomastoideus	+	++ (Dysphagia)		Carotid artery
Longus colli/capitis	+++	+++		Carotid artery, jugular vein, vagal nerve, phrenic nerve
Scalene muscles	+++	+++	Levator scapulae	Brachial plexus, external jugular vein
Levator scapulae	++	+	Scalenus posterior, splenius capitis (lower part), Trapezius	Lung apex
Splenius capitis	+ (Injection often too deep)	++ (weakness of neck extensors)	Semispinalis, longissimus, obliquus capitis inferior	Might be frequently very thin during continuous treatment
Longissimus capitis/cervicis	+++	++	Splenius capitis, semispinalis, levator scapulae	Vertebral artery
Semispinalis capitis/cervicis	+	+	Splenius capitis, obliquus capitis inferior, Trapezius	
Obliquus capitis inferior and small neck extensors	+++	+++	Semispinalis capitis, other small neck muscles	Vertebral artery, N. occipitalis major, spinal canal
Trapezius	+		Levator scapulae; splenius capitis, Semispinalis	Might be frequently very thin, especially during continuous treatment

The application of US as injection control avoids unintended muscle weakness due to diffusion of BoNT A into adjacent muscles and in consequence reduces the incidence of adverse reactions. This is especially important in cervical dystonia, as neck muscles might be small or thin and lie in close proximity to each other. Hong et al. 2012 report five cases of preselected patients who met criteria for cervical dystonia and subsequent dysphagia after EMG-guided injections. Dysphagia could be completely eliminated in these patients by addition of ultrasound guidance. The same might be especially true for avoidance of unintended weakness of neck extensor muscles.

US is also indispensable when case-specific anatomic conditions are present such as obesity or very pronounced neck muscles. In addition, muscle atrophy can occur in consequence of previous treatments and even muscles like the splenius capitis or trapezius, which are usually injected without imaging guidance, can become very thin, thus putting the attending physician at risk to inject too deep.

Furthermore, the application of US helps to prevent injection errors in blood vessels and nerves as summarized in Table 1. Compared to injections in the extremities like in spasticity treatment, this might be of special importance in CD as injections in lateral cervical muscles bear a high risk for injuries of adjacent anatomical structures (e.g., carotid artery, thyroid truncus, internal and external jugular vein, vagal nerve, phrenic nerve, and brachial plexus). With injections in the dorsal neck region such as the obliquus capitis inferior muscle, the vertebral artery or the spinal canal can be erroneously injected. Furthermore, sonography-guided injections may reduce the bleeding risk in patients receiving anticoagulant treatment.

Besides, anatomically precise injection of typically treated superficial muscles, US guidance enables injection of up to now not routinely treated deep cervical muscles, which contribute to a broad range of neck movements and are involved in complex forms of cervical dystonia (Bhidayasiri 2011). These deep muscles include prevertebral muscles for primary neck flexion, lateral vertebral muscles for shoulder elevation, neck and head tilt, and a posterior group of muscles for neck extension (Fig. 1c, d). Some complex forms of cervical dystonia with involvement of deep cervical muscles are only poorly accessible by routine BoNT A therapy. Table 1 provides an overview of the relevance of US for location of different muscles, which can be affected in patients with cervical dystonia.

The implementation of more differentiated clinical concepts distinguishing between individual contributions of the distinct neck and head muscles (-collis/-caput concept, Reichel 2011) gives rise to the need of a visualization method for precise differentiation and localization of the target muscle. The successful realization of this more realistic phenomenological approach is only feasible with highly controlled and precise US-guided injections.

Moreover, US guidance offers the potential for dose reduction and a decrease of the total number of injections/injected muscles—factors that are potentially reducing the risk of producing neutralizing antibodies and therefore ensure long-term efficacy of BoNT A treatment in cervical dystonia.

Finally, US guidance ensures a highly standardized injection procedure resulting in a stable effect, better comparability between different injection sessions, and consistency in case of change of the treating physician. This circumstance is of special importance for cervical dystonia patients being treated for many years.

### US for anatomical knowledge

A secondary but important effect of using US is the improvement of anatomical knowledge of the injecting physician. This is especially meaningful and indispensable for beginners who need to be trained in the specific and complex anatomy of neck muscles. However, consolidation of anatomical knowledge by applying US is also reasonable for experienced therapists. Finally, even patients who are usually injected without US guidance may profit from improved anatomical understanding of the treating physician.

### Disadvantages of US

Currently, there are only a few practical drawbacks. The immediate availability of ultrasound equipment is not always and everywhere guaranteed, and there is a lack of reimbursement, especially in comparison to EMG. The additional expenditure of time for application of US can be reduced by training programs.

### Clinical studies and future perspectives

In general, efficacy of BoNT A treatment in cervical dystonia is high (e.g., Costa et al. 2005; Hallett et al. 2013). Nevertheless, there is a potential for further improvements, and refined clinical and diagnostic concepts warrant prospective clinical studies. As an optimized BoNT A treatment is based on (a) accurate identification of involved muscles and (b) accurate injection of identified muscles, US guidance should be included in future studies to ensure standardized injection procedures as well as proper targeting of identified muscles. Although diagnostic application of US for muscle identification is not within the scope of this review, US might be used for detection of muscle hypertrophy. Furthermore, the combination of US and EMG could overcome the shortcomings of EMG regarding anatomic precision and would allow an accurate assignment of dystonic or tremulous activity to specific muscles (Schramm et al. 2014).

## Conclusions and expert statement

US is an increasingly available, non-invasive, and inexpensive method, which has clear advantages over other techniques for injection control in BoNT A treatment of cervical dystonia. It facilitates anatomically precise and reproducible injections in specific muscles, therefore carrying the potential of enhancing efficacy and safety of BoNT A treatment in cervical dystonia. It is a valuable method to improve anatomical knowledge and the quality of future clinical studies.

The implementation of US guidance in clinical routine will also lead to an improvement of BoNT A injection schemes and our functional understanding in cervical dystonia, because only a precise injection in the target muscles allows judgement of the relevance of the respective muscles for the dystonic pattern. US therefore facilitates the correct identification of the functionality of muscles in relation to patient phenomenology. A valid concept of the role of different muscles in multidimensional movement disorders can only be developed if there is no doubt which muscles have really been injected or at least if the uncertainty can be reduced as far as possible. This has been pointed out by Reichel (2011) in connection with the -caput/-collis concept using CT, but is applicable for sonography, as this method can be used repeatedly in patients without putting them at risk of radiation.

Therefore, from the perspective of the authors there is no convincing reason why US guidance for injection control should not become standard in BoNT A treatment of cervical dystonia.

In conclusion, due to the advantages of US-guided BoNT A injections and the experience of the authors in clinical practice, the authors summarize their opinion as follows:

- Beginners should learn the specific anatomy of neck muscles and exact targeting of BoNT A treatment by application of US.
- Expertise in US guidance should be available at each BoNT A treatment center.
- In the opinion of the authors, the routine use of US injection guidance could be recommended in general.
- Preferably, US guidance should be applied in the following cases:
  - Presence of individual anatomic conditions (pronounced neck muscles, atrophy, and obesity)
  - Occurrence of adverse events after BoNT A injections (especially dysphagia and unintended weakness)
  - Presence of complex dystonic patterns with a high probability of involvement of deeper located muscles which are more difficult to access (see Tab. 1; relevance ++/+++).
  - Secondary non-responders without neutralizing antibodies against BoNT A
- US guidance should be included in future clinical studies.
